# Transmission of quantum-secured images

**DOI:** 10.1038/s41598-024-62415-2

**Published:** 2024-05-21

**Authors:** Steven Johnson, John Rarity, Miles Padgett

**Affiliations:** 1 School of Physics and Astronomy, University of Glasgow, Glasgow, G12 8QQ UK; 2 Department of Physics, University of Strathclyde, Glasgow, G4 0NG UK; 3 Quantum Engineering Technology Labs, H. H. Wills Physics Laboratory, Department of Electrical and Electronic Engineering, University of Bristol, Bristol, BS8 1FD UK

**Keywords:** Optics and photonics, Physics

## Abstract

The secure transmission of an image can be accomplished by encoding the image information, securely communicating this information, and subsequently reconstructing the image. Alternatively, here we show how the image itself can be directly transmitted while ensuring that the presence of any eavesdropper is revealed in a way akin to quantum key distribution (QKD). We achieve this transmission using a photon-pair source with the deliberate addition of a thermal light source as background noise. One photon of the pair illuminates the object, which is masked from an eavesdropper by adding indistinguishable thermal photons, the other photon of the pair acts as a time reference with which the intended recipient can preferentially filter the image carrying photons from the background. These reference photons are themselves made sensitive to the presence of an eavesdropper by traditional polarisation-based QKD encoding. Interestingly the security verification is performed in the two-dimensional polarisation-basis, but the image information is encoded in a much higher-dimensional, hence information-rich, pixel basis. In our example implementation, our image comprises of 152 independent pixels. Beyond the secure transmission of images, our approach to the distribution of secure high-dimensional information may offer new high-bandwidth approaches to QKD.

## Introduction

The transmission of information using a quantum-secure protocol is becoming of major importance in encrypted communications. Techniques based on quantum key distribution (QKD) are set to become mainstream for the defence and finance industries to securely send encryption keys and know if the information has been intercepted, albeit one limitation is the low speed of data transmission^1,2^. In parallel to advances in this communication technology, advances in quantum imaging are using correlations between photons to enable images to be obtained in noisy regimes^3,4^. Bringing these two technologies together to securely encode photons in a high dimensional basis offers new ways of transmitting images and may also increase the data capacity of a communication system.

A polarisation encoding system based upon QKD BB84 uses the polarisation state of the light to encode a photon in a binary state corresponding to one bit per photon^5^. As with all approaches to QKD, key is that this polarisation information can be encoded in at least two mutually unbiased measurement bases, in this case, vertical:horizontal (V:H), or diagonal:anti-diagonal (D:A), or, right-circular:left-circular (R:L) polarisations. The sender and intended recipient transmit and measure single photons in a random series of these bases and subsequently compare their results. An eavesdropper can intercept these photons but when they re-transmit they must also randomly select a basis, and in doing so inevitably make different choices from the recipient and therefore corrupt the channel, hence revealing their presence. The requirement for single photon operation, inherent transmission/detection losses, and various necessary error corrections mean that the secure bit rate is much smaller than a classical, albeit insecure, system.

The transmission of information using polarisation states for encryption has become a standard technique that underpins the rapid development of quantum communications. Higher dimensional quantum secure communication has also been studied^6,7^. These high dimensional approaches again are centred on mutually unbiased measurement bases such as time:frequency^8^, orbital angular momentum:angle^9^, and position:transverse momentum^10^. There has also been recent interest in the transmission of secure images, as a basis for high dimensional communications^11–13^.

More generally, images as a means of encrypted communication has long been a method of communicating securely by hiding information within an image, and had been done digitally in a large number of applications^14,15^. The sending of images with correlated photons works in a similar way to ghost imaging^16–19^. The importance of transmitting quantum secure communications in environments with background light has also been shown to be important for its development, such as the transmission of QKD signals within daylight^20^.

In this work we take a different approach to encryption that builds upon previous work for hiding low intensity images in background noise. This earlier work used a photon-pair source producing time-correlated signal and idler photons. The signal beam was used to create an image using a programmable mask and the idler beam was used to trigger a detector, this triggered enabled the spatial position of the signal photons to be recorded and hence, summed over many photons, the image. This image was hidden from casual eavesdroppers by deliberately adding background light to the image, hence only a recipient with access to the idler photons to trigger the camera could isolate the true image from the background noise^21^. This system in itself gives some element of security, but in principle an eavesdropper could intercept the trigger photons, use them to extract their own image and then re-transmit both the image and the trigger photons to the intended recipient. In this present work we overcome this limitation by polarisation encoding the trigger photons and hence, inspired by QKD-BB84, ensuring that the presence of any eavesdropper is revealed.

Within our previous work we showed that an image could successfully be hidden within the shot noise fluctuations of a constant background signal, this was achieved using a 2-photon source, where one photon was sent in the image arm and the other photon sent to the receiver as a heralding signal, where time-gating was used to successfully record the respective photon within the image arm. If an eavesdropper had accessed the heralding signal they would be able to reconstruct the image and there would be no encryption. To secure this heralding arm the QKD protocol is proposed to ensure this heralding arm, and hence the image arm, is preserved. The experimental realisation of the QKD secured system is shown in this work.

Interestingly, in our approach, although the information is recorded in a high-dimension Hilbert space (i.e. position) the security is ensured by a traditional QKD-inspired approach within a two-dimensional space (i.e. polarisation). In essence the trigger idler photons are secured by their polarisation to give temporal information, with which to extract the spatial information of the time-correlated signal photon. Inherent to our security is the need for background noise being added to the information, i.e. the noise in the system is a virtue not a limitation. This need for noise makes our approach particularly suited to situations where the background noise is inherent to the operation, such as optical transmission in free-space.

## Methodology

**Figure 1:**
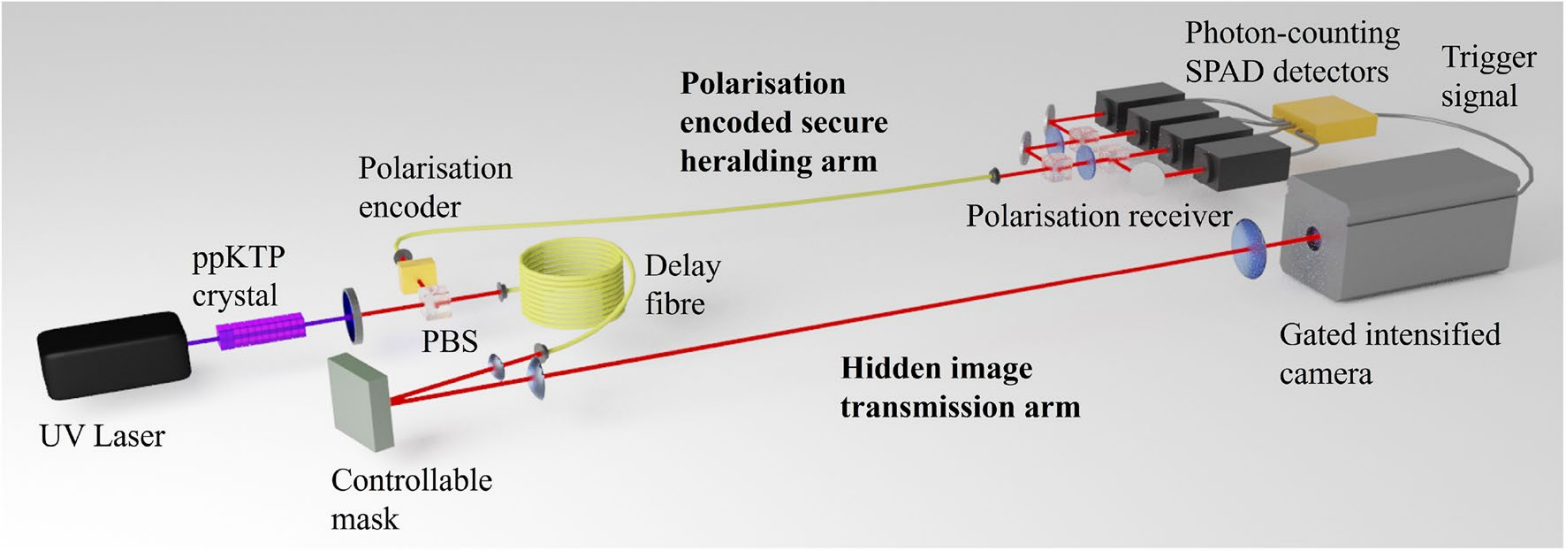
An illustration of the experimental set up. The UV laser passing through the ppKTP crystal produced a photon-pair. The polarisation state of the idler photon was set by a half-wave plate and transmitted through an optical fibre to a polarisation detection scheme with a SPAD detector for each polarisation state, the signals from the detectors were summed and output to trigger the intensified camera. The signal photon was transmitted through the imaging arm, where the image was created on a programmable mask and imaged to the gated intensified camera. The image was hidden from a casual eavesdropper by the deliberate addition of thermal noise photons.

The core of our system was the spontaneous parametric down conversion (SPDC) source, the photon-pairs from which were separated using a polarisation beam splitter into both the QKD heralding arm (idler) and the image transmission arm (signal). An experimental schematic is shown in Fig. 1. The SPDC source was a ${405}\,\textrm{nm}$ diode laser pumping a ppKTP type-II non-linear crystal with ${9.8}\,\upmu \textrm{m}$ polling and ${30}\,\textrm{mm}$ in length. After the crystal, the UV pump beam was removed with a long-pass wavelength filter. The signal and idler beams had orthogonal polarisation and could be separated using a polarising beam splitter (PBS) and coupled into optical fibres^22^.

The heralding arm used a rotatable half-waveplate to set the polarisation of the light to be horizontal (H), vertical (V), diagonal (D) or anti-diagonal (A). The light was coupled into a ${2.8}\,\textrm{m}$ single-mode optical fibre, where a manual fibre polarisation controller was used to ensure the polarisation was faithfully transmitted through to the exit of the optical fibre. The receiver used a removable beam-splitter to make a choice for the measurement basis of the heralding photon. On one output was a PBS to measure the H and V polarisation states, on the other was a half-waveplate to rotate the polarisation by 45^∘^ and a PBS to measure the D and A polarisation states. The photon measurements were made using single-photon avalanche diode (SPAD) photon-counting modules (Excelitas SPCM-AQRH). Each channel used a SPAD detector, and the counts were recorded using a 4-channel time-to-digital converter (quTAG). The quTAG enabled the individual count rates to be measured and the alignment of the system to be optimised. The photon-pair source produced 250,000 counts-per-second for the heralding signal, which could be transmitted in any of the polarisation bases. The signals from the four SPAD detectors were summed using a 4-channel OR-gate (SN74LVC32AD), where the output of the OR-gate was then used to trigger the camera in the imaging arm.

In the image transmission arm the photons first passed through a long single-mode optical fibre (${50}\,\textrm{m}$) that acted as a delay-line to ensure the image carrying photons arrived at the camera slightly after the heralding photons. The light from the optical fibre was collimated and illuminated a digital micro-mirror device (Vialux V-7001), which acted as a programmable mask to generate the image to be transmitted to the camera. The image was then transmitted ${1.8}\,\textrm{m}$ through free space and imaged onto a gated intensified CCD camera (Andor iStar 334T).

The gated intensified camera took an image when triggered by the polarisation encoded heralding arm. The digital delay within the camera was set such that the temporally correlated photon was captured during the ${10}\,\textrm{ns}$ activation of the gated intensified camera. The camera was read out every 0.1 s and the frame stored. In our system the heralding efficiency was 5%, however the quantum efficiency of the gated intensified camera at the operation wavelength was low ($<1\%$), meaning that there was a very low efficiency of detection of the heralded photons, but these technological limitations would be easily overcome with different hardware.

The image signal was hidden from a casual observer by adding additional background light, this was achieved using an LED at the signal wavelength (${800}\,\textrm{nm}$), bandpass-filtered to be indistinguishable from the signal image carrying photons. The LED was computer controlled and calibrated to give a known level of background light.

Inspired by BB84, the prepared polarisation state of the heralding photons was transmitted, and at the receiver a random choice of measurement basis was made. Comparing the polarisation states before and after optical transmission it was possible to identify if an eavesdropped had been accessing the heralding channel.

## Results

Whenever a heralding signal was received the camera would be triggered to collect the time-correlated photons. For example, when the H polarisation state was sent and the measurement basis was H:V it would be expected to see approximately 100% of the counts on the H channel and 0% on the V channel, when the measurement basis was A:D then one expected approximately 50% on both the D channel and A channel. If an eavesdropper intercepted and re-transmitted the heralding photons, then one would have seen a deviation from this distribution. Of particular significance would be a deviation from 0% of measurements made in a state orthogonal to the state of the transmitted photon.

**Figure 2:**
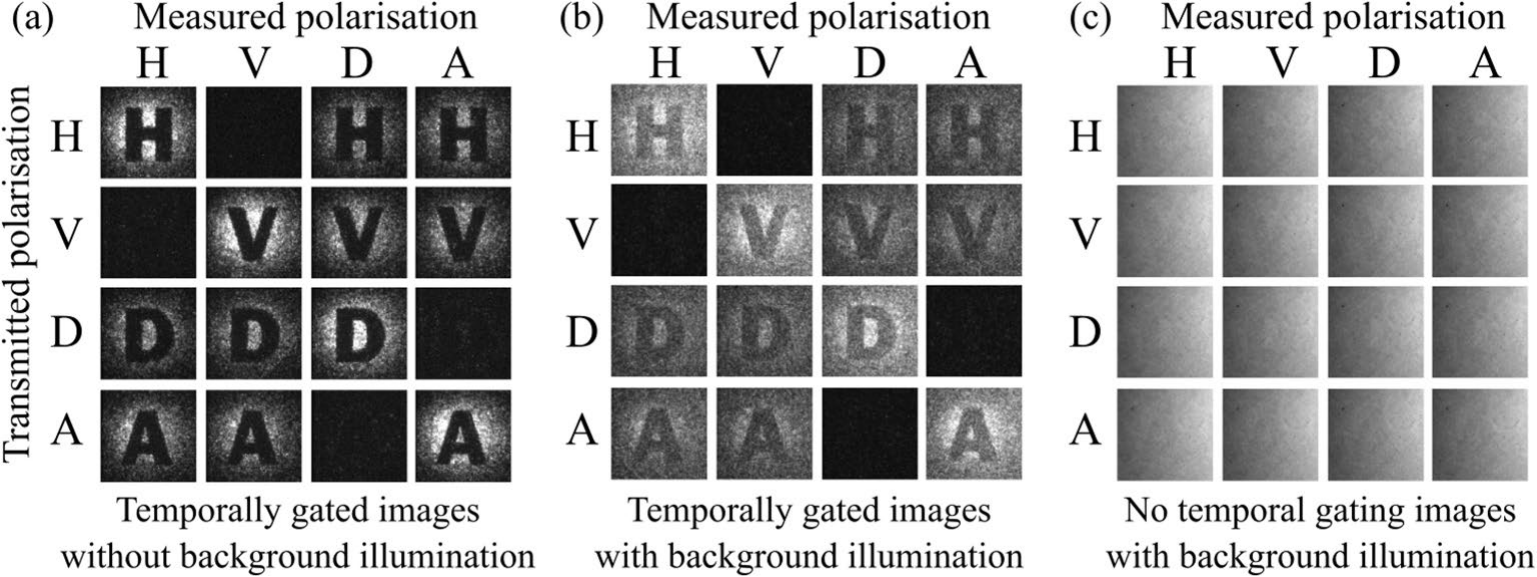
(**a**) The image captured on the camera for a 30 second measurement is shown the horizontal (H), vertical (V), diagonal (D) and anti-diagonal (A) polarisation states, where the letter in the transmitted image is the transmitted polarisation state. The images were measured with no background light and using the trigger from a single detection channel to temporally gate the image. (**b**) With the addition of background light the transmitted images are still seen when using temporal gating. (**c**) With the addition of background light and temporal gating is not used, the camera images are dominated by the background illumination and the images are not identifiable.

A corresponding set of images are shown in Fig. 2a, where the camera was triggered directly by the relevant H, V, D or A channel detector. The transmitted polarisation state in the heralding arm is indicated by the letter used in the image projection of that state, and the measured image for each of the polarisation channels is shown. Adding background noise to the imaging channel would cause the transmitted image to be obscured, such that a casual eavesdropper would be unable to detect the image without having access to the heralding signal. In the temporally gated images, Fig. 2b, a ${10}\,\textrm{ns}$ exposure was used when each trigger was received, the transmitted images were recorded with only marginal amounts of additional noise from the background illumination in the image. Whereas when there is no gating used, Fig. 2c, the images become hidden within the shot-noise of the high background signal.

**Figure 3:**
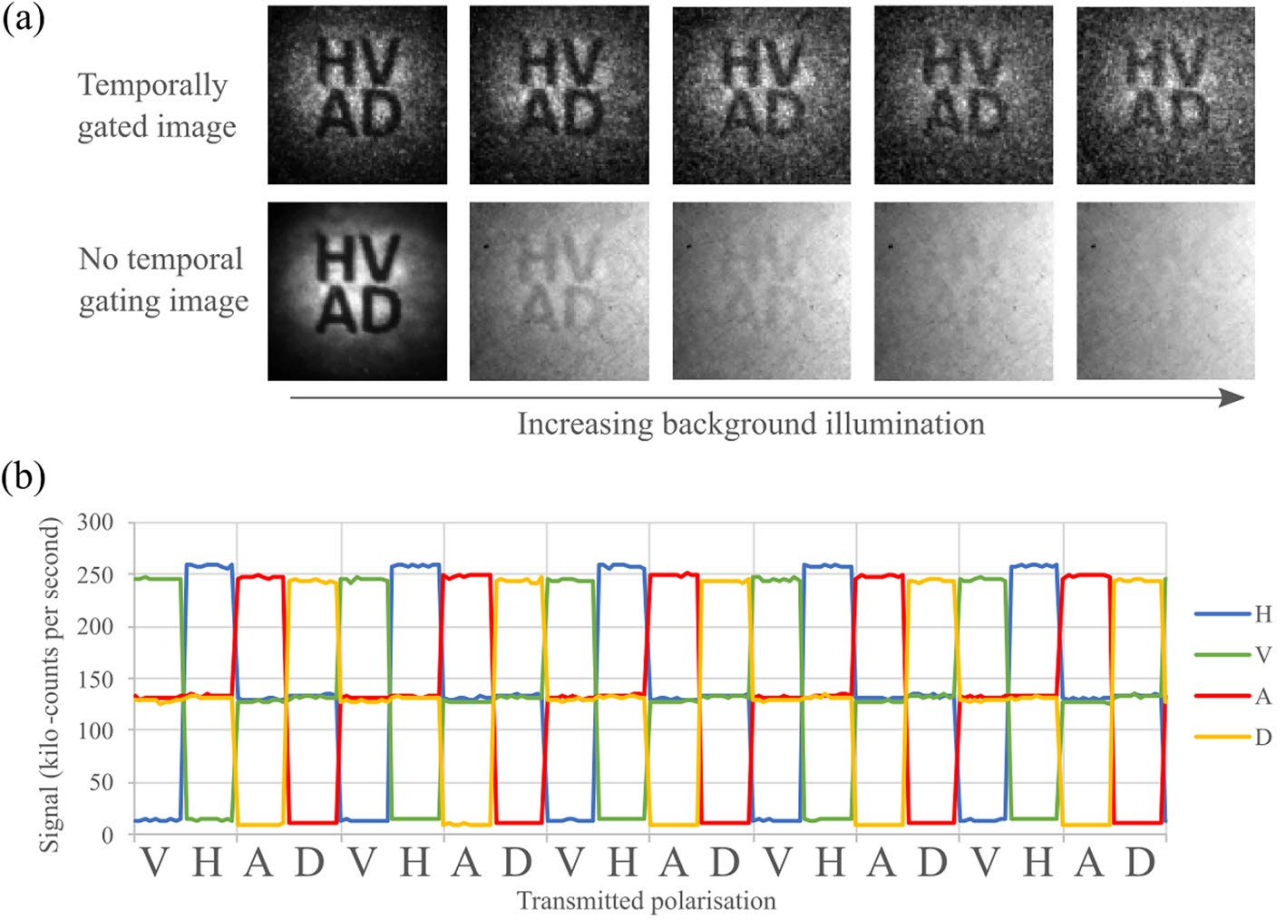
(**a**) The images shown used the combined triggers from all four polarisation channels (HVDA). The projected image is obscured by increasing the background light level. (**b**) The signal measured in each of the receiver polarisation channels changed depending on the transmitted polarisation state. The signal shown was measured with the photon counting detectors while the images were recorded on the camera.

To demonstrate how the heralding signal from all channels could be used, images were acquired with varying amounts of background noise, both with and without gating. The heralding signals from each of the SPAD detectors were summed together with an OR-gate. The visibility of the image is seen for a range of background illumination intensities as shown in Fig. 3a. The signal count measured for the individual channels during a period of image acquisition is shown in Fig. 3b, where the transmission channel alternated between polarisation states while the signal measured on each receiver channel was measured.

**Figure 4:**
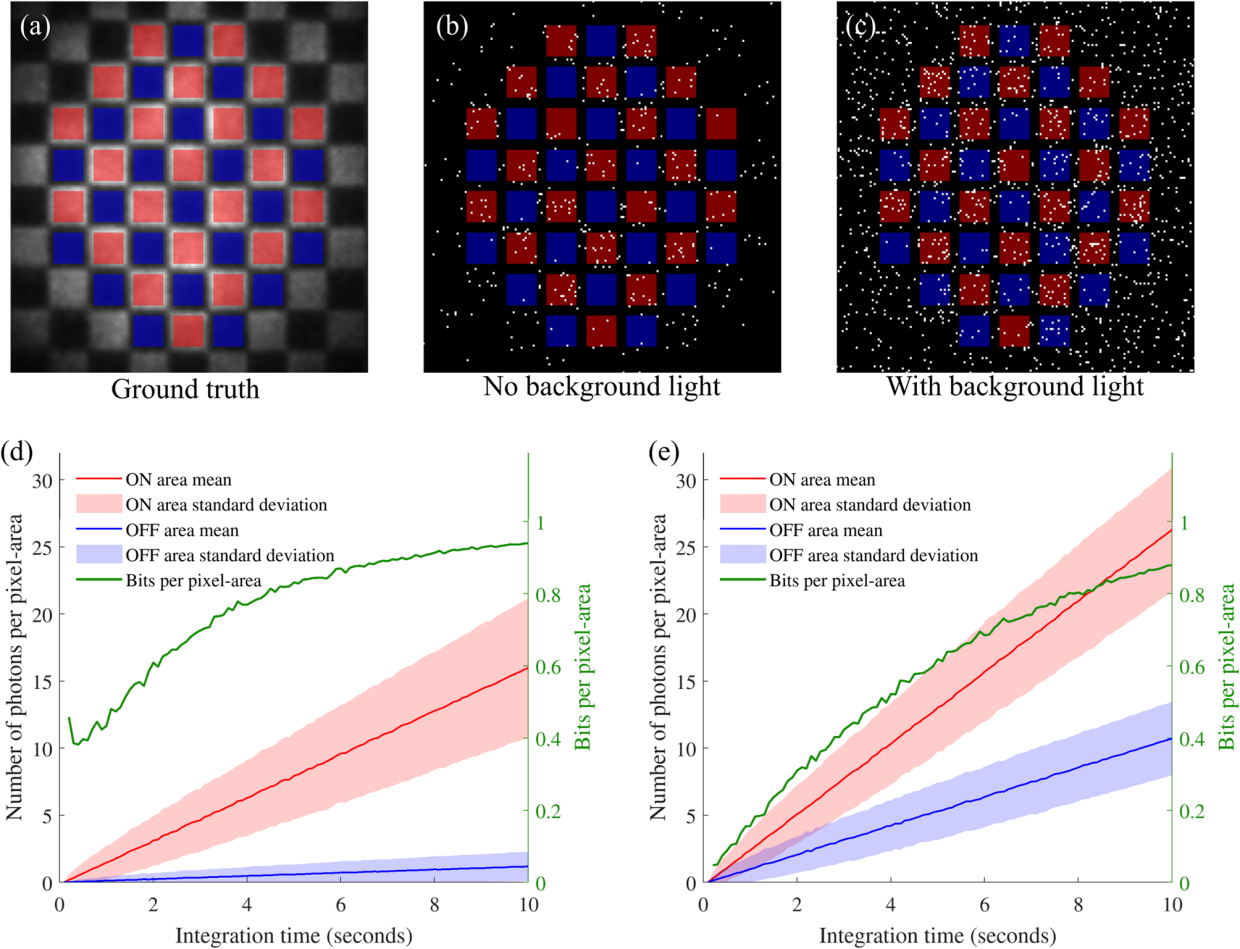
(**a**) The ground truth image of the projected checkerboard, overlaid are red squares denoting the area of the ON pixels and blue squared denoting the area of the OFF pixels (non-gated long exposure image). (**b**) A image of the photon-counting events acquired over 5 s with no background illumination, a greater number of photon event occur within the ON pixel areas. (**c**) A image of the photon-counting events acquired over 5 s with high background illumination, the difference between the ON an OFF pixel-areas is less clear, with a greater number of photon counts due to the background light. (**d**) A plot showing the number of photons measured in the ON (red) and OFF (blue) pixel-areas for exposure times ranging between 0.1 and 10 s, with the associated bits per pixel-area. (**e**) A plot showing the number of photons measured in the presents of a high background illumination, the ON and OFF areas are shown to be distinguishable at times greater than 5 s, where the variation on pixel-area values overlap.

The limitation of the data readout would be the indistinguishability of the signal arriving at the pixel-areas. To ascertain when a signal would be detectable within set pixel-areas a checkerboard was projected on to the camera, the projected image was made up of areas where the programmable mask was ON and OFF. A ground-truth image was acquired from 10 seconds of non-gated exposure, Fig. 4a, from this image the regions relating to ON (shown in red) and OFF (shown in blue) within the central part of the illumination beam are overlaid. A total of 23 ON areas and 23 OFF areas were identified within the image. In a transmission system these pixel-areas would be toggled within the projected image to transmit data in a binary sequence. Temporally gated images with an acquisition time of 0.1 s were taken and analysed to measure the photon numbers in each pixel-area, where up to 100 frames were summed together. The arrival of heralded photons at the intensified camera would appear as a cluster of signal across a few pixels in the raw images, the centre of each cluster was found and the position assigned as a single photon event. The images captured for 5 s of exposure are shown in Fig. 4b, c, where the images had no background illumination and a high background illumination respectively. The photon events in each ON area and OFF area were summed to calculate the signal per pixel-area. The average number of counts for each pixel-area (the mean of the 23 pixel-areas) for both the ON and OFF averaged over 100 measurements, along with the standard deviation of the pixel-areas and bits per pixel-area, is shown in Fig. 4d. Where background noise had been added the number of photons in both the ON and OFF areas increased, as shown in Fig. 4e. As can be seen there was little crossover between the signal (above 1 s) when there was no background light, when the background light was added there was significant crossover; it would be unclear if the pixel-area transmitted was ON or OFF. For an observer without the heralding signal (the non-gated image) the background photon number would be 1,000,000 times higher for both the ON and OFF, significantly hiding the image signal. The bits per pixel-area is calculated from the binary Shannon entropy, which is the likelihood of recovering the wrong bit from a measurement of a pixel-area. This has been calculated from the normal distributions for the ON and OFF pixel-areas to give probability *p* of measuring the wrong bit. The binary entropy function *H*(*p*) calculates the entropy, where the bits per pixel-area is $1-H(p)$^23^, as plotted in Fig. 4d, e.

The measurements were repeated for the ON and OFF areas using squares of half the size, in this arrangement there are 76 ON areas and 76 OFF areas (totalling 152 areas). The time required to have a clear separation in the ON and OFF areas was around four times longer, as would be expected for squares half the size containing four times less flux of photons. The data for this result can be found in the data repository.

Rather than transmitting graphical images, the images can be defined as a binary digital matrix e.g. an $8\times 8$ checkerboard, the individual squares representing a 0 or 1, i.e. the image overall being a representative of a 64-bit number. We see that in our implementation, in the presence of background noise to hide the image, this the ability to distinguish ON from OFF is maintained for integration times above 5 s, corresponding to approximately 500 detected photons per image.

If we were to adopt this methodology purely for the transmission of high dimensional data, one approach would be to replace the programmable transmission mask with a beam steering component (e.g. high-speed galvo scanner) to direct the photons to one of the 64 squares, thereby encoding a 6-bit number. This would decrease the loss as only half of the light is sent to the receiver when a controllable mask is used and would also reduce the intrinsic losses associated with using a controllable mask. All the photons would be transmitted when using beam steering, thereby reducing the signal-to-noise in the resulting image measurement.

## Discussion

We have demonstrated that images produced from a time-correlated photon-pair source can be hidden within a background of optical noise. The polarisation state encoding allows a post-measurement comparison of states between the sender and receiver to ensure the heralding photons were sent securely, c.f. the well-known BB84 QKD protocol. The imaging arm achieves covertness by hiding the image photons such that they cannot be differentiated from the optical background via any temporal, wavelength or polarisation filtering. This transmission of images can be used for the covert communication of information in a secure way.

Although the information is recorded in a high-dimensional Hilbert space the security is ensured by a traditional QKD-inspired approach within a 2-dimensional space (i.e. polarisation). In essence the heralding idler photons are secured by their polarisation to give temporal information, from which to extract the spatial information of the time-correlated signal photon. In the case of free-space communications there would be background noise present within the measurement, this already existing background noise could be used to conceal the signal.

A possible method of attack on the security of our approach would exploit the fact that timing and polarisation are not inextricably linked. In principle the heralding photon could be intercepted, its arrival time extracted and then re-transmitted with the same, and by necessity unmeasured, polarisation state. One potential way of achieving this would be by quantum teleportation, where the quantum state of an input photon, which is destroyed in the process, can be copied to the outgoing photon^24^. Central to this copying process is the Bell-state analyser, the coincidence count from which would reveal the timing information whilst not collapsing the polarisation state. However presently available Bell-state analysers rely on the indistinguishability of photons and the use of an entangled photon source, itself synchronised to (or heralded by) the incoming photon. Practically speaking either of these aspects leads to the analyser having a low probability of success and a dramatic reduction in photon rate or errors in the post analysis of the polarisation data revealing the eavesdropper. To address this security weakness, it might be possible to secure the heralding channel by using time-bin encoding protocols such as COW, meaning that a potential eavesdropper would disturb the coherence between successive time-bins revealing their presence^25^.

Whilst we demonstrate the principle of our approach, we acknowledge that our specific implementation does not yet reach the data-rate of any existing and optimised traditional QKD system. We also note that our approach could be improved by a more efficient pattern formation and by deliberately imposing a time-varying pattern on our background light. However, none of these limitations should undermine what could be a new concept in QKD, namely one where high-dimensional channel is hidden in noise and then secured using more traditional techniques in a lower dimensional space.

## Data Availability

The raw image data generated from the image acquisition and the analysis code that support the findings of this study are available from the University of Glasgow Enlighten repository with the identifier https://doi.org/10.5525/gla.researchdata.1613.
